# Supplementary material for: Quantitative live-cell imaging and computational modeling shed new light on endogenous WNT/CTNNB1 signaling dynamics
Source: eLife. 2021 Jun 30;10:e66440. doi: 10.7554/eLife.66440 (PMC8341982; doi:10.7554/eLife.66440)
Supplement: Transparent reporting form [file elife-66440-transrepform.docx]

***eLife’s* transparent reporting form**

We encourage authors to provide detailed information *within their submission* to facilitate the interpretation and replication of experiments. Authors can upload supporting documentation to indicate the use of appropriate reporting guidelines for health-related research (see [EQUATOR Network](http://www.equator-network.org/%20)), life science research (see the [BioSharing Information Resource](https://biosharing.org/)), or the [ARRIVE guidelines](http://www.plosbiology.org/article/info:doi/10.1371/journal.pbio.1000412) for reporting work involving animal research. Where applicable, authors should refer to any relevant reporting standards documents in this form.

If you have any questions, please consult our Journal Policies and/or contact us: [editorial@elifesciences.org](mailto:editorial@elifesciences.org).

**Sample-size estimation**

- You should state whether an appropriate sample size was computed when the study was being designed
- You should state the statistical method of sample size computation and any required assumptions
- If no explicit power analysis was used, you should describe how you decided what sample (replicate) size (number) to use

Please outline where this information can be found within the submission (e.g., sections or figure legends), or explain why this information doesn’t apply to your submission:

- Sample sizes were not computed, but decided based on practical considerations and experience with the experimental techniques performed.

**Replicates**

- You should report how often each experiment was performed
- You should include a definition of biological versus technical replication
- The data obtained should be provided and sufficient information should be provided to indicate the number of independent biological and/or technical replicates
- If you encountered any outliers, you should describe how these were handled
- Criteria for exclusion/inclusion of data should be clearly stated
- High-throughput sequence data should be uploaded before submission, with a private link for reviewers provided (these are available from both GEO and ArrayExpress)

Please outline where this information can be found within the submission (e.g., sections or figure legends), or explain why this information doesn’t apply to your submission:

- Number of measurements and number of individual biological replicates are indicated in all figure legends or accompanying tables.
- The definition of the technical replicates for qRT-PCR and luciferase assays is provided in the methods section.
- For Western Blot, luciferase assay, qRT-PCR and N&B no data were excluded.
- For live imaging, inclusion and exclusion of segmented cells (based on the size of the segmented cytoplasm) is described in the method section “Time-lapse imaging”
- For FCS measurements, inclusion and exclusion of data (based on bleaching and/or large intensity fluctuations) is described in the methods section “FCS data acquisition and analysis”.

**Statistical reporting**

- Statistical analysis methods should be described and justified
- Raw data should be presented in figures whenever informative to do so (typically when N per group is less than 10)
- For each experiment, you should identify the statistical tests used, exact values of N, definitions of center, methods of multiple test correction, and dispersion and precision measures (e.g., mean, median, SD, SEM, confidence intervals; and, for the major substantive results, a measure of effect size (e.g., Pearson's r, Cohen's d)
- Report exact p-values wherever possible alongside the summary statistics and 95% confidence intervals. These should be reported for all key questions and not only when the p-value is less than 0.05.

Please outline where this information can be found within the submission (e.g., sections or figure legends), or explain why this information doesn’t apply to your submission:

- Calculation of inferential statistics (e.g. 95% confidence intervals and P-values) is described in the methods section “Data representation and statistical analysis”.
- Raw data points are shown throughout, with the exception of Figure 3, where the large number of datapoints would not be informative to display.
- Not all P-values are reported in the main text, but they have been included for FCS and N&B parameters (Figure 5, 7, 8 and accompanying supplements).
- Supplementary File 1 provides a complete overview of the median, mean and 95% CI’s contains for all FCS and N&B measurements. Relevant P-values have been included in this file as well.

(For large datasets, or papers with a very large number of statistical tests, you may upload a single table file with tests, Ns, etc., with reference to sections in the manuscript.)

**Group allocation**

- Indicate how samples were allocated into experimental groups (in the case of clinical studies, please specify allocation to treatment method); if randomization was used, please also state if restricted randomization was applied
- Indicate if masking was used during group allocation, data collection and/or data analysis

Please outline where this information can be found within the submission (e.g., sections or figure legends), or explain why this information doesn’t apply to your submission:

- Samples were not randomized.
- Experimental groups were defined based on control versus treatment conditions. Individual samples/wells were allocated into different experimental groups based on a logical order during setup and execution of the experiment.

**Additional data files (“source data”)**

- We encourage you to upload relevant additional data files, such as numerical data that are represented as a graph in a figure, or as a summary table
- Where provided, these should be in the most useful format, and they can be uploaded as “Source data” files linked to a main figure or table
- Include model definition files including the full list of parameters used
- Include code used for data analysis (e.g., R, MatLab)
- Avoid stating that data files are “available upon request”

Please indicate the figures or tables for which source data files have been provided:

***Raw unprocessed data:***

- Figure 1 and supplements: original FACS data have been uploaded as .fcs files on Open Science Framework (<https://osf.io/dczx8/>)
- Figure 2 and supplements: original Western blot scans have been uploaded on Open Science Framework (<https://osf.io/dczx8/>)
- Figure 3 and Figure 8 supplement 1: original live cell confocal imaging data have been uploaded as .tif files on Open Science Framework (<https://osf.io/dczx8/>).
- Figure 5, 7 and 8: original FCS traces have been uploaded as .ptu/.sptw files and .oif files (reference image with ROI of the measuring point) on Open Science Framework (<https://osf.io/dczx8/>). Note: example traces and images depicted in Figure 4 were also extracted from these files.
- Figure 5, 7 and 8: original N&B data have been uploaded as converted .tif stacks (together with downstream ImageJ analyses and non-normalized results) and .oif files (reference image for subcellular localization) on Open Science Framework (<https://osf.io/dczx8/>).
- Figure 7 supplement 1: original FACS data have been uploaded as .fcs files on Open Science Framework (<https://osf.io/dczx8/>)
- Figure 7 supplement 1: original Western blot scans have been uploaded on Open Science Framework (<https://osf.io/dczx8/>)
- A full description of the computational model, including a full list of the parameters used, is provided in the material and methods section “Model description”.
- A full description of the FCS fitting model, including a full list of the parameters used, is provided in the materials and methods section “FCS data acquisition and analysis”.

***Source data:***

- A comprehensive overview of all numerical data (summary statistics; median, mean and 95% CI’s) for the FCS and N&B experiments depicted in Figures 5, 7, 8 plus accompanying supplements and in Tables 1, 2 and 3) is provided in summary tables as Supplementary File 1.
- Figure 2 + supplements: source data are provided in .xlsx format
- Figure 3 + supplements: source data for all graphs in .xlsx format
- Figure 5 + supplements: source data for all graphs in .xlsx format
- Figure 7 + supplements: source data for all graphs in .xlsx format
- Figure 8 + supplements: source data for all graphs showing experimental data in .xlsx format

***Code for data analysis:***

- Scripts for the following have been made publicly available on Open Science Framework (<https://osf.io/dczx8/>), as referenced in the materials and methods section:
- Cell profiler segmentation pipeline (Figure 3)
- R script based on PlotsOfDifference to generate Figure 3 supplement 2 and supplementary movies 4-6
- ImageJ N&B analysis script (Figures 5,7 and 8)
- R source code for the computational model (Figure 6)
